# Electrophysiological mapping of the epicardium via 3D‐printed flexible arrays

**DOI:** 10.1002/btm2.10575

**Published:** 2023-07-19

**Authors:** Terrence Pong, Kevin J. Cyr, Cody Carlton, Joy Aparicio‐Valenzuela, Hanjay Wang, Meghedi Babakhanian, Alessandro Maiuolo, Haley Lucian, Paul J. Wang, Y. Joseph Woo, Anson M. Lee

**Affiliations:** ^1^ Department of Cardiothoracic Surgery School of Medicine, Stanford University Stanford California USA; ^2^ Department of Cardiovascular Medicine School of Medicine, Stanford University Stanford California USA

**Keywords:** 3D printing, atrial fibrillation, cardiac ablation, flexible electronics, in vivo cardiac mapping, Langendorff perfusion, ventricular tachycardia

## Abstract

Cardiac electrophysiology mapping and ablation are widely used to treat heart rhythm disorders such as atrial fibrillation (AF) and ventricular tachycardia (VT). Here, we describe an approach for rapid production of three dimensional (3D)‐printed mapping devices derived from magnetic resonance imaging. The mapping devices are equipped with flexible electronic arrays that are shaped to match the epicardial contours of the atria and ventricle and allow for epicardial electrical mapping procedures. We validate that these flexible arrays provide high‐resolution mapping of epicardial signals in vivo using porcine models of AF and myocardial infarction. Specifically, global coverage of the epicardial surface allows for mapping and ablation of myocardial substrate and the capture of premature ventricular complexes with precise spatial–temporal resolution. We further show, as proof‐of‐concept, the localization of sites of VT by means of beat‐to‐beat whole‐chamber ventricular mapping of ex vivo Langendorff‐perfused human hearts.

## 1 INTRODUCTION

Three‐dimensional (3D) printing technologies have been deployed in multiple domains of medicine, including the development of presurgical planning models, bioprinted tissues, and more recently, patient‐specific devices and implants.^1^, ^2^ The field of cardiac surgery is particularly suited to benefit from these advances as illustrated by recent advances in the development of 3D‐printed heart valves.^2^ These patient‐specific approaches recognize the individual anatomical differences that exist between patients to move beyond the one‐size‐fits‐all approach of existing medical technology. This is especially beneficial in the field of cardiac electrophysiology and arrhythmia surgery,^3^ where patient‐specific tools may be used to advance the success of cardiac ablation.^2^

Heart rhythm disorders, such as atrial fibrillation (AF), impact over 33 million people globally with a prevalence that continues to rise as the population ages.^4^ These conduction disorders often have a structural basis caused by tissue remodeling that varies between individual patients.^5^, ^6^ Catheter ablation has emerged as a valuable treatment for patients who fail to respond to anti‐arrhythmic drugs; yet, the localization of AF substrate remains a significant challenge. Current interventions for AF have focused on identifying and disrupting pathogenic foci within the heart. However, interventions have limited success, with ~50% of patients experiencing recurrence of AF following initial catheter ablation.^6^

Catheters represent one of the most widely used technologies for the capture of electrograms and delivery of energy to targeted sites within the myocardium.^7^ However, catheter‐based approaches suffer from limited spatial density, require frequent repositioning, and carry a small but potentially life‐threatening risk of cardiac perforation.^8^, ^9^ Strategies to improve the accuracy and safety of catheter procedures have included the use of noninvasive electrocardiographic imaging (ECGi), mapping with 3D electroanatomic registration systems, and epicardial mapping with the off‐label use of endocardial catheter probes,^10^ each with significant limitations in their ability to effectively target regions of arrhythmia.^11^, ^12^, ^13^ New diagnostic and mapping approaches that can more accurately evaluate arrhythmogenic sites and offer precise localization of disease foci may lead to improved surgical intervention and therapeutic success.^14^, ^15^

The ability to map the entire myocardial surface at once may prevent discrepancies introduced during sequential mapping and help identify novel substrates of arrhythmia that are missed during mapping of focal areas. Recent advancements in the field of flexible, thin‐film electronics have created the opportunity to build dynamic mapping tools for the epicardial surface.^16^, ^17^, ^18^, ^19^, ^20^ Here, we leverage these techniques to present a translational approach to deploy epicardial mapping within the cardiac surgical environment. We describe the use of preoperative models in combination with 3D printing to construct flexible mapping devices that capture unique patient‐specific morphology and optimize electrode contact with the epicardial surface of the heart. The specific platform reported here allows for conformal contact with the epicardial surface and provides the option for concurrent delivery of energy for the purpose of electrical stimulation and radiofrequency (RF) ablation (RFA). The devices are deployed and tested in the cardiac surgical environment for their ability to aid surgeons in arrhythmia interventions.^21^

## 2 RESULTS

### 2.1 Flexible device design and fabrication

**FIGURE 1:**
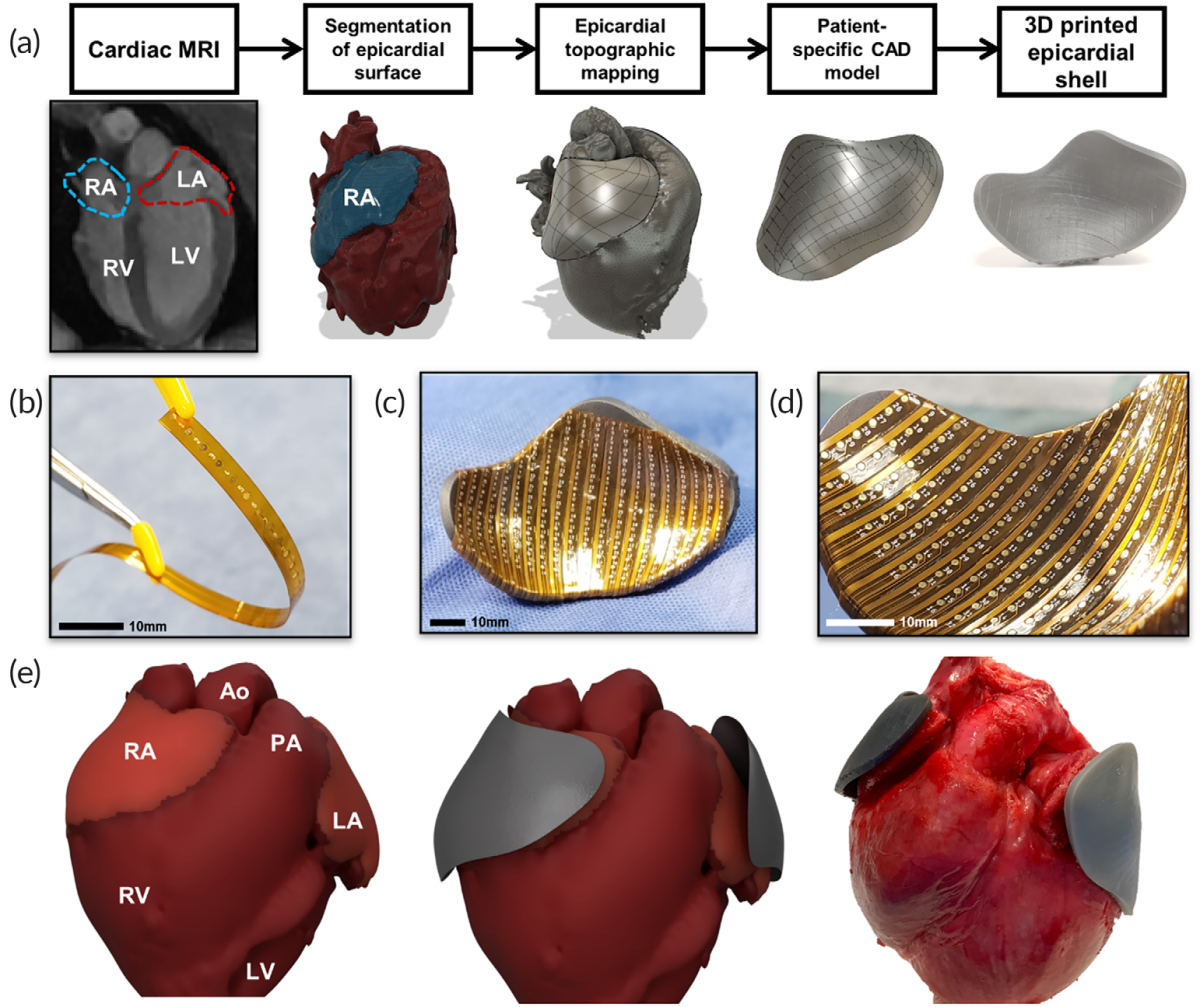
Patient‐specific flexible electrode array design and characterization. (a) Example workflow to create mapping array for the right atrium (RA). A cardiac MRI was performed to obtain detailed whole‐chamber resolution of the myocardium. Heart chamber segmentation generated a 3D model of the epicardial surface with the RA highlighted in blue. A computer‐aided design (CAD) model was generated that matched the anatomic features of the epicardial surface, which was then used to 3D print a flexible epicardial shell. (b) Image of flexible linear electrode array, interelectrode spacing was optimized in the preoperative CAD environment. (c) Electrode arrays were attached to the surface of the epicardial shell to create a patient‐specific array capable of whole‐chamber epicardial mapping. (d) Mapping electrodes adopted the geometry of the epicardial surface to optimize signal acquisition. (e) (left) 3D model of the whole heart depicting the segmentation of the RA, (center) presurgical CAD rendering of the flexible epicardial shells and, (right) postsurgical photo of the ex vivo porcine heart overlaid with custom whole‐chamber flexible array. Ao, aorta; LA, left atrium; LV, left ventricle; PA, pulmonary artery; RA, right atrium; RV, right ventricle.

The design of 3D epicardial arrays began with cardiac magnetic resonance imaging (MRI) utilizing late‐gadolinium enhancement. Epicardial models of the atria and ventricle were obtained from end‐diastolic MRI image data and threshold‐based segmentation.^22^ Representative segmentation of the right atrium (RA) shown in Figure 1a, demonstrates classification of the surface of interest. The segmented epicardial surface was then used to render a computer‐aided design (CAD) model, and a one‐to‐one scale epicardial shell was printed with a commercial stereolithography 3D printer using flexible photopolymer resin.

The 3D‐printed shell served as a substrate for the attachment of custom‐designed, flexible electrode arrays (Figure 1b). In order to provide optimal signal acquisition across the whole chamber, the interelectrode spacing and electrode orientation were organized in the preoperative CAD environment prior to manufacture. To complete fabrication, the electrode arrays were attached to the surface of the epicardial shell to create a flexible array with patient‐specific 3D geometry (Figure 1c). The final system allowed for conformal contact with the epicardial surface (Figure 1d). The completed patient‐specific electrode array was then connected to a biopotential measurement system for signal acquisition.

Critical checkpoints for the production of the patient‐specific arrays are highlighted in Figure 1e, beginning with MRI segmentation of the chambers of interest (left panel). Followed by CAD of patient‐specific shells to capture epicardial topography (center) and final comparison of the 3D‐printed electrode array with the ex vivo reference heart (right). Each patient‐specific device required <24 h to manufacture, starting from image acquisition to final assembly. Future translational research may benefit from these rapid production workflows to create individualized arrays for clinical and research applications.

### 2.2 Electromechanical mechanics during cardiac cycle

**FIGURE 2:**
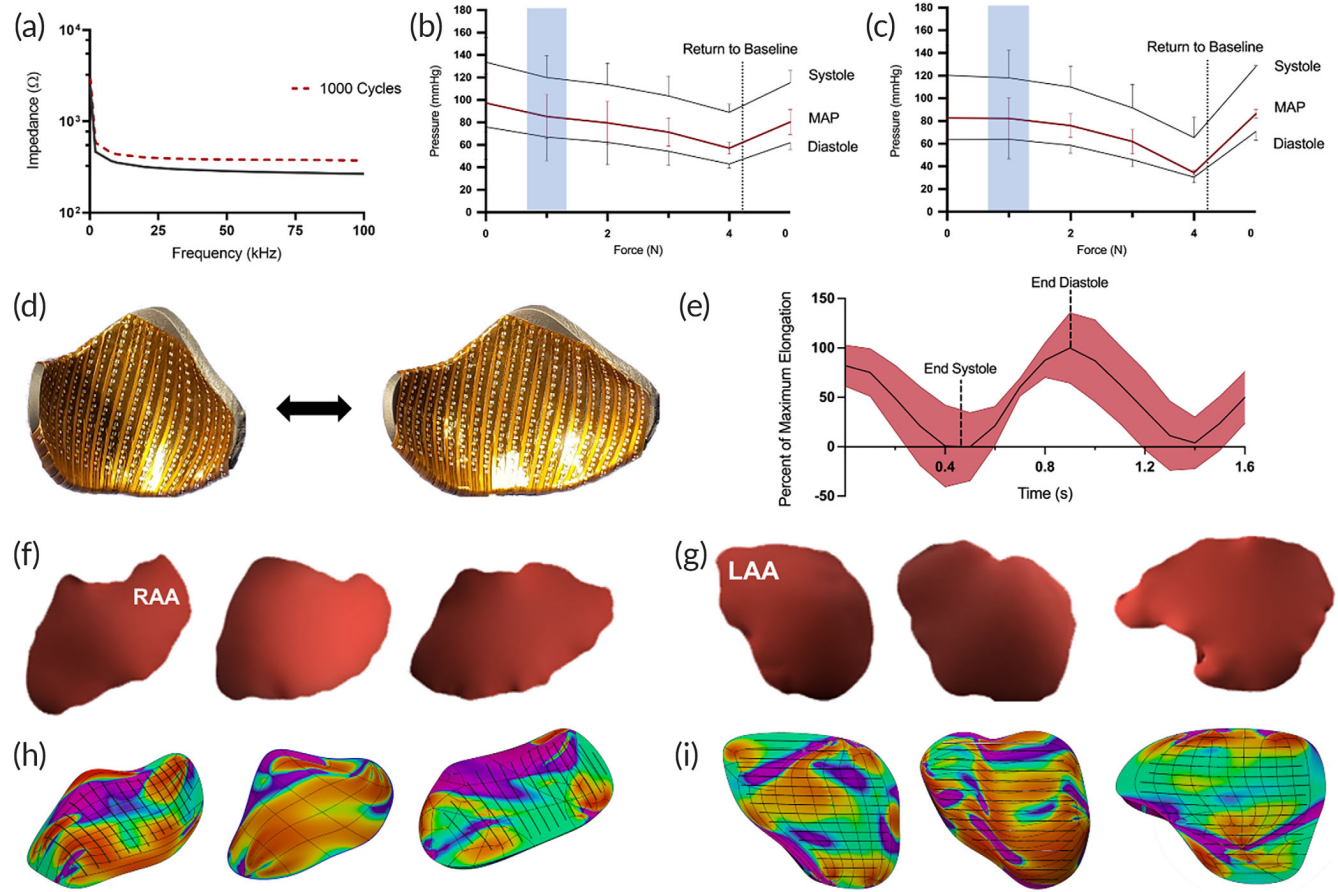
Electromechanical characterization of 3D printed flexible whole‐chamber epicardial arrays. (a) Impedance spectrum of flexible electrodes after 1000 cycles of ±50% strain (b,c), in vivo characterization of relationship between systemic mean arterial blood pressure (MAP) and external force applied by the epicardial array over the right and left atrium, respectively. The region highlighted in blue indicates the range of force (1 ± 0.15 N) necessary to ensure proper electrode contact with the epicardium. Forces above 4 N caused significant hemodynamic compromise. (d) Epicardial arrays were 3D printed with flexible photopolymer resin allowing for dynamic adaptation to changes in epicardial morphology over the course of a cardiac cycle. (e) Strain analysis during the cardiac cycle demonstrated percentage deformation of the device over the period of a cardiac cycle (red, ±1 SD). Analysis of atrial anatomy of different hearts demonstrates a high degree of morphological variability. (f,g) Anterior view of right and left atrial segmentations obtained from three different cardiac MRIs. (h,i) Gaussian curvature analysis of respective right and left atrial epicardial shells highlight the degree of topographical variability between individuals. LAA, left atrial appendage; RAA, right atrial appendage.

A crucial requirement for successful epicardial mapping is consistent electrode contact throughout the duration of the cardiac cycle. A key benefit of 3D printing is the broad range of materials with variable flexural strength available for additive manufacturing. In order to maintain a consistent electromechanical interface between electrodes and the epicardium, we utilized a flexible photopolymer resin with the ability to maintain shape after many strain cycles. Mechanical characterization of the tensile properties of the 3D printed shells with sensors attached revealed a Young's modulus of 7.5 ± 0.4 MPa, yield strength of 1.66 ± 0.03 MPa, and ultimate strength of 2.3 ± 0.04 MPa (*n* = 6). Figure 2a depicts characterization of impedance performance of the flexible electrode arrays in 0.9% NaCl solution after 1000 cycles of repeated ±50% bending strain in the direction parallel to the long‐axis of the linear arrays. No statistical difference in impedance performance was observed before and after cyclic testing, 31.8 ± 7.7 Ω vs. 31.0 ± 3.8 Ω (*p* = 0.48) and 58.5 ± 13.6 Ω vs. 60.1 ± 18.5 Ω (*p* = 0.42) at 1 kHz and 10 kHz, respectively (*n* = 10).

To assess the impact of the flexible epicardial arrays on cardiovascular hemodynamics, we performed arterial blood pressure measurements during contact with in vivo porcine hearts via median sternotomy. Integrated capacitive force sensors were used to provide quantitative analysis of the forces necessary to achieve complete electrode contact with the epicardium. The patient‐specific geometry allowed for close conformation with the native epicardial surface, and 1 ± 0.15 N of force was sufficient to ensure contact with all electrodes (*n* = 3). We observed that mean arterial pressure (MAP) was more readily affected by the application of external cardiac force to the lower‐pressure atrial chambers while application of force to the higher‐pressure ventricular chambers did not affect systemic MAP. There was minimal change in MAP when 1 N of force was applied on the right and left atrium (LA; Figure 2b,c). Application of higher forces, above those needed for normal electrogram acquisition, indicated that forces >4 N caused hemodynamic instability resulting in MAPs below 60 mmHg. The results, based on invasive in vivo characterization suggest that the forces required to perform electrophysiological mapping using epicardial devices are safe and do not cause significant hemodynamic compromise.

Next, we characterized the degree of flex sustained by the patient‐specific arrays over the course of a cardiac cycle by performing strain analysis in vivo. The flexible devices were designed to match the epicardial contours of the atria and ventricle, and the natural flexibility allowed for dynamic adaptation to changes in anatomic conformation of the underlying epicardium (Figure 2d). We observed deformation of the flexible arrays with up to 50% contraction during systole and 50% elongation during diastole along the long axis (Figure 2e). The flexible arrays were capable of performing continuous electrogram acquisition in excess of 1000 cycles without signal degradation, and we observed no crack formation or delamination of electrodes along the bending zones.

### 2.3 In vivo porcine cardiac anatomic measurements

Healthy and arrhythmogenic hearts can exhibit a high degree of morphological variability in the human population. The anatomy of the right and left atria are of critical importance when considering interventional procedures for catheter ablation. Using the methods described, we fabricated individualized flexible arrays to perform epicardial mapping in healthy and diseased (chronic AF) adult Yorkshire swine. Similar to humans, we observed high variability in atrial morphology, with the antero‐lateral portion of the RA dominated by the right atrial appendage (RAA) and high variations in RAA morphology. Examples of variable atrial morphology from different pigs are shown in Figure 2f,g. The left atrial appendage (LAA) was characterized by finger‐like projections from the main body of the LA, with similarly high variations in morphology (Figure 2g). Renderings of the epicardial shell designs and subsequent Gaussian curvature analysis highlight the degree of variable epicardial morphology between individual atria (Figure 2h,i). The pseudocolor maps depict regional curvature differences between individual epicardial shells of the right and left atrium, respectively. Deeper blue colors represent more negative Gaussian curvature (hyperbolic) and more red colors represent positive Gaussian curvature (spherical).

### 2.4 Atrial measurements of normal sinus rhythm, stimulation, and ablation

**FIGURE 3:**
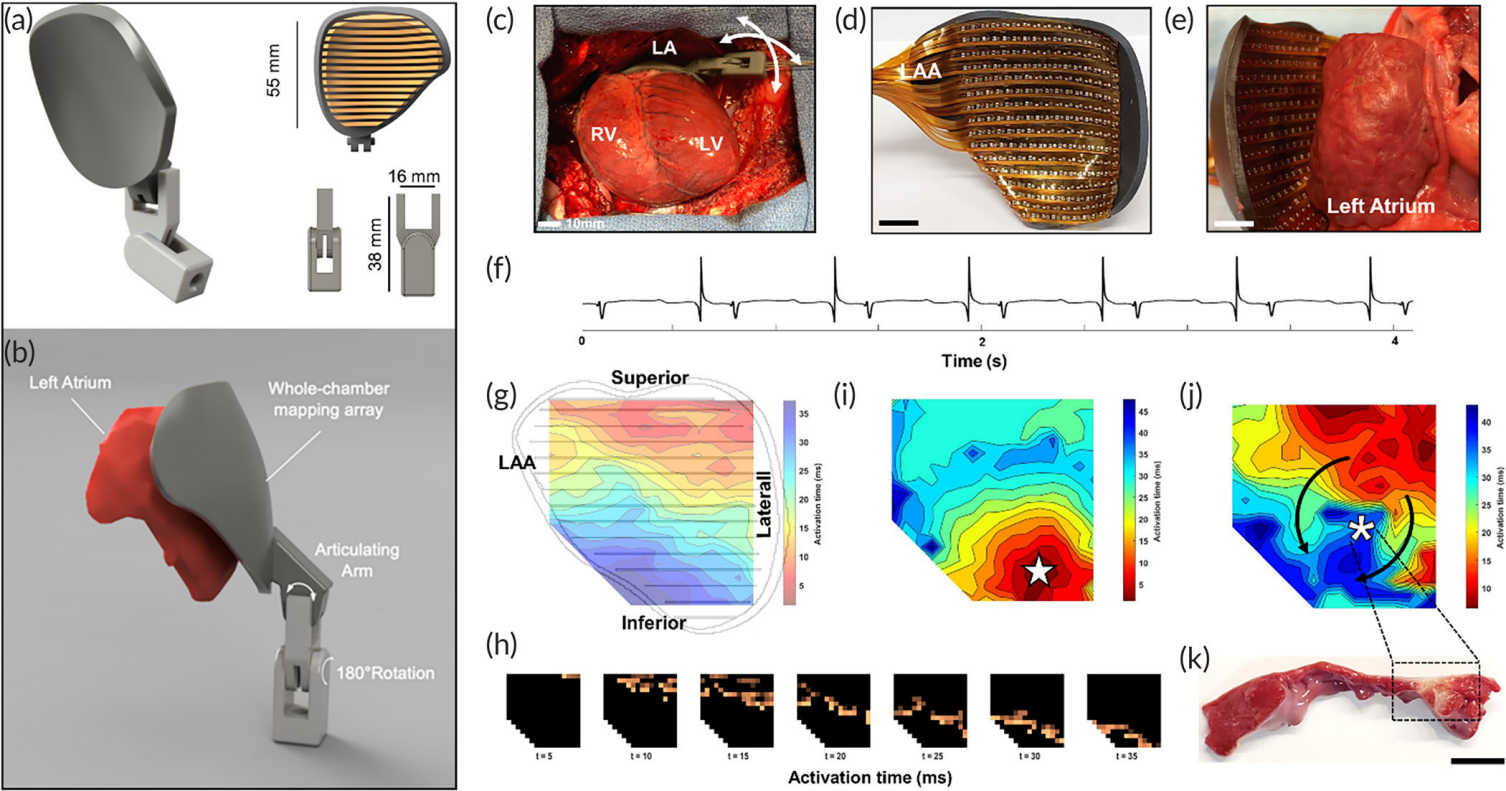
In vivo cardiac mapping, pacing, and ablation with whole‐chamber electrode arrays. (a) CAD rendering of multiaxis articulating arm for positioning of whole‐chamber mapping arrays. (b) Articulating arms facilitate placement of the mapping array on the epicardial surface. (c) Surgical deployment of the mapping array over the left atrium via median sternotomy. (d) Example high‐density (16 × 16) flexible electrode array for the left atrium. Scale bar, 10 mm. (e) Demonstration of geometric conformity between the flexible array and LA epicardial surface. Scale bar, 10 mm. (f) Representative unipolar left atrial electrogram from the flexible array. (g) Isochrone activation map during sinus rhythm determined by (dV/dt)_max_. (h) Time‐lapse visualization of individual electrode activation at 5 ms time intervals demonstrates sequential action potential wave propagation during sinus rhythm. In addition to electrophysiological mapping, the electrode arrays were capable of delivering focused energy for the purpose of extra stimulus pacing and RF ablation. (i) Isochronal activation during extra stimulus pacing via two adjacent electrodes demonstrated retrograde action potential propagation (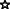
denotes pacing location). (j) RF energy was delivered through electrodes located at the center of the flexible array followed by postablation isochrone activation mapping. Black arrows indicate the direction of wave front propagation around site of thermal injury, (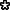, denotes location of RF ablation). (k) Image of ablation lesion after triphenyltetrazolium chloride staining. Scale bar, 5 mm. LA, left atrium; LAA, left atrial appendage; LV, left ventricle; RF, radiofrequency; RV, right ventricle.

To validate the function of the patient‐specific flexible arrays, we performed in vivo cardiac mapping of healthy adult porcine hearts. Adult swine were anesthetized and underwent median sternotomy to expose the anterior surface of the heart. Delivery of the mapping system was facilitated by integration with an articulating multiaxis pivot joint placed at the end of a cylindrical instrument shaft. A CAD rendering of a mapping array for the left atrium is shown in Figure 3a,b, with in vivo deployment of the system shown in Figure 3c. The completed whole‐chamber left atrial mapping device, as depicted in Figure 3d, demonstrates the conformal alignment of the mapping array with the left atrial surface (Figure 3e). Demonstration of flexible array mapping of the left atrium is shown in Video S1. A representative left atrial voltage trace from the electrode array during normal sinus rhythm is shown in Figure 3f. Isochronal analysis of electrogram activation during sinus rhythm demonstrates the action potential originating from the superolateral aspect of the LA free wall and propagating toward the inferomedial border Figure 3g. The estimated conduction velocity was 1.04 ± 0.28 ms^−1^ at an average heart rate of 114 ± 8.2 beats/min (*n* = 5). Figure 3h depicts individual electrode activation at 5 ms time‐intervals determined by (d*V*/d*t*)_max_ of unipolar signal (where *V* is electrogram signal and *t* is time).

Differences in anatomy between human and swine precluded mapping of the posterior pulmonary veins.^23^ However, exposure and epicardial mapping of the pulmonary veins in humans during cardiac surgery has been previously shown to be feasible and safe.^24^, ^25^, ^26^ We anticipate that the workflows developed here can be readily adapted for epicardial mapping of the pulmonary veins in humans.

In addition to cardiac mapping, we explored the potential for the flexible arrays to deliver energy for the purpose of: (1) epicardial pacing and (2) RF ablation. Normal sinus rhythm was observed to originate from the superolateral aspect of the LA free wall, so external pacing was applied in a location away from this normal activating pattern. The epicardium was overdrive paced with bipolar stimulation applied through two adjacent electrodes along the inferior left atrial border, with subsequent epicardial activation captured with the flexible array. Pacing of the inferior border resulted in activation originating between the two paired electrodes (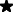), followed by a propagation in a retrograde, inferior to superior fashion, Figure 3i. Whole‐chamber coverage using flexible arrays enabled customization of energy vector delivery through selection of individual paired electrodes, which may be useful for advanced diagnostic electrophysiological procedures in the future.

Next, to assess the ablation capabilities of the flexible arrays, RF lesions were produced with a standard RF lesion generator. RF energy was delivered through two adjacent electrodes for 30 s, followed by electrophysiological mapping with the same array. The flexible arrays captured epicardial activity immediately before and after the delivery of RF energy, demonstrating concomitant mapping and ablation capabilities. Action potential characterization postablation indicated wave propagation around the thermal lesion (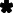), Figure 3j. Subsequent staining with triphenyltetrazolium chloride (TTC) showed transmural ablation of the atrial myocardium, Figure 3k. The high‐resolution electrode density of the flexible array allowed for the precise delivery of external pacing energy and RF energy, and energy delivery could be customized by choosing different electrodes without moving the array.

This tailored approach may prove beneficial in situations where manual steering of a catheter tip is difficult or time‐consuming.^27^ Taken together, we show that the flexible arrays introduced here can serve as a robust, rapid prototyping platform for in vivo mapping, external stimulation, and ablation, which are fundamental to the current paradigm for clinical diagnosis and treatment of arrhythmias.

### 2.5 Epicardial mapping of chronic AF

**FIGURE 4:**
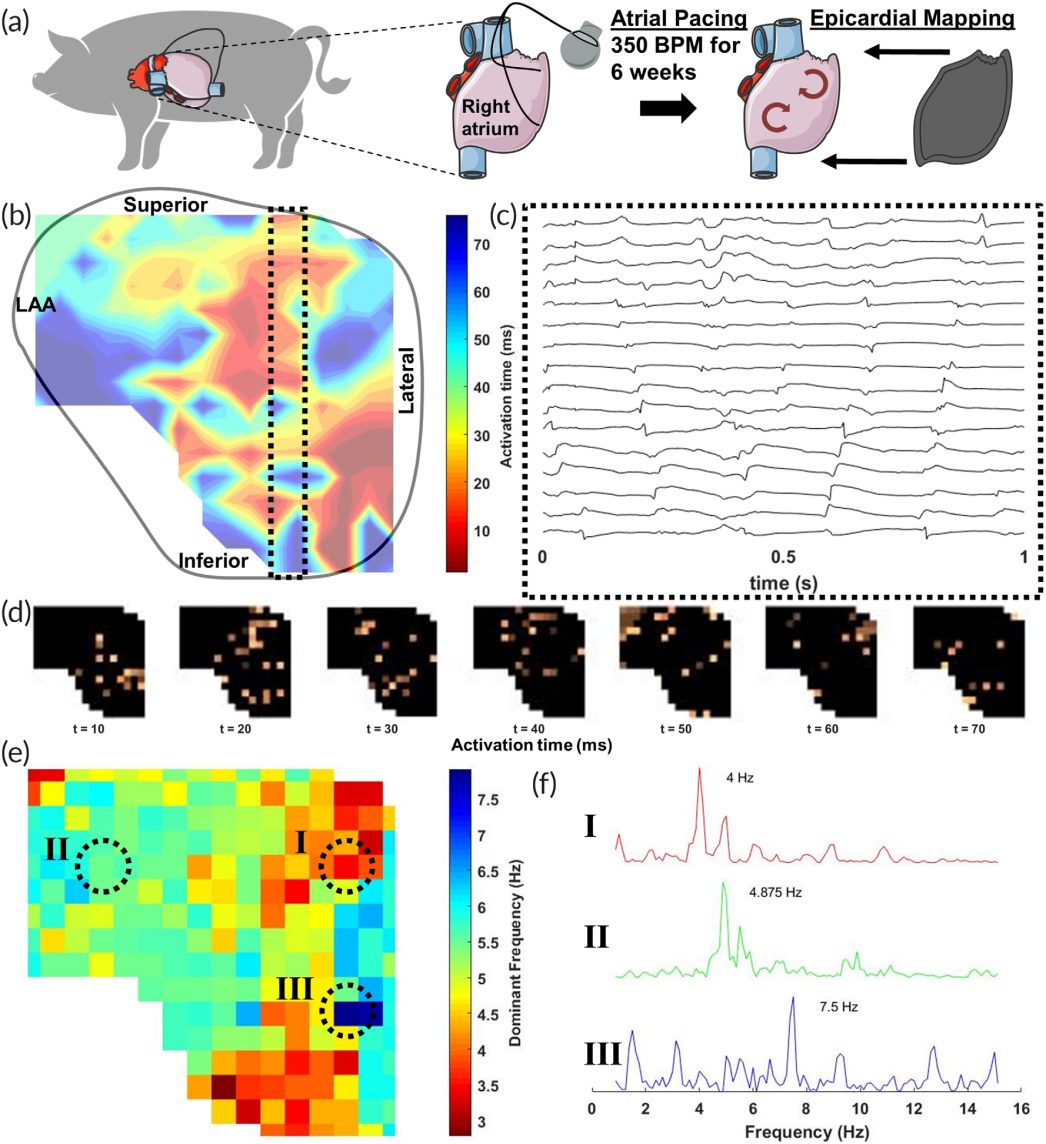
Cardiac mapping of the left atrium during chronic AF. (a) Schematic showing pacing‐induced chronic AF in a porcine model. Yorkshire swine underwent right atrial pacing at 350 beats/min for 6 weeks prior to terminal cardiac mapping. (b) Isochrone activation maps demonstrate random fibrillatory activity and loss of coordinated action potential wave propagation when compared to sinus rhythm consistent with atrial fibrillation. (c) Representative electrograms from the black dashed box in (b) are characterized by low‐amplitude and high‐frequency signals. (d) Time‐lapse visualization of individual electrode activation at 10 ms time intervals demonstrates random fibrillatory pattern of atrial activation. (e) Frequency domain visualization of individual electrode dominant frequency (DF). (f) Representative power spectrums of three electrodes identified in e with varying DF.

To assess the performance of the flexible arrays in an arrhythmogenic setting we employed a porcine model of chronic AF as previously described.^28^, ^29^ The discordant firing of atrial substrate during AF resulted in signals of significantly lower amplitude compared to those during normal sinus rhythm, which can be difficult to characterize.^30^ Yorkshire swine underwent pacemaker insertion, followed by continuous rapid atrial pacing for 6 weeks to induce chronic AF, Figure 4a. Isochrone analysis of AF electrograms demonstrates the loss of coordinated action potential wave propagation, replacement with multiple sites of simultaneous activation, and fragmented areas of local depolarization, Figure 4b. Representative electrograms derived from the dashed box in Figure 4b, depict low‐amplitude, high‐frequency fractionated electrograms characteristic of AF, Figure 4c.^31^ Similarly, (d*V*/d*t*)_max_ visualization of electrode activation illustrates loss of sequential activation as seen in sinus rhythm, replaced by a random fibrillatory pattern of epicardial activation, Figure 4d.

Frequency domain analysis further quantified the characteristics of the electrograms during chronic AF.^32^, ^33^ Whole‐chamber mapping enabled high‐resolution characterization of dominant frequency (DF) changes in chronic AF, Figure 4e. Example power spectra for points I, II, and III are shown in Figure 4f. In this case, the DF distribution is notable for the presence of maximal DF along the posterolateral border of the left atrium (dashed circle III). Our results show successful in vivo recording of arrhythmogenic atria activity in a large animal model using patient‐specific arrays to record global atrial activity. Whole‐chamber resolution, beat‐to‐beat mapping of AF signaling may help identify novel substrates of arrhythmia that are missed during conventional mapping of focal areas.

### 2.6 Ventricular mapping following myocardial ischemia

**FIGURE 5:**
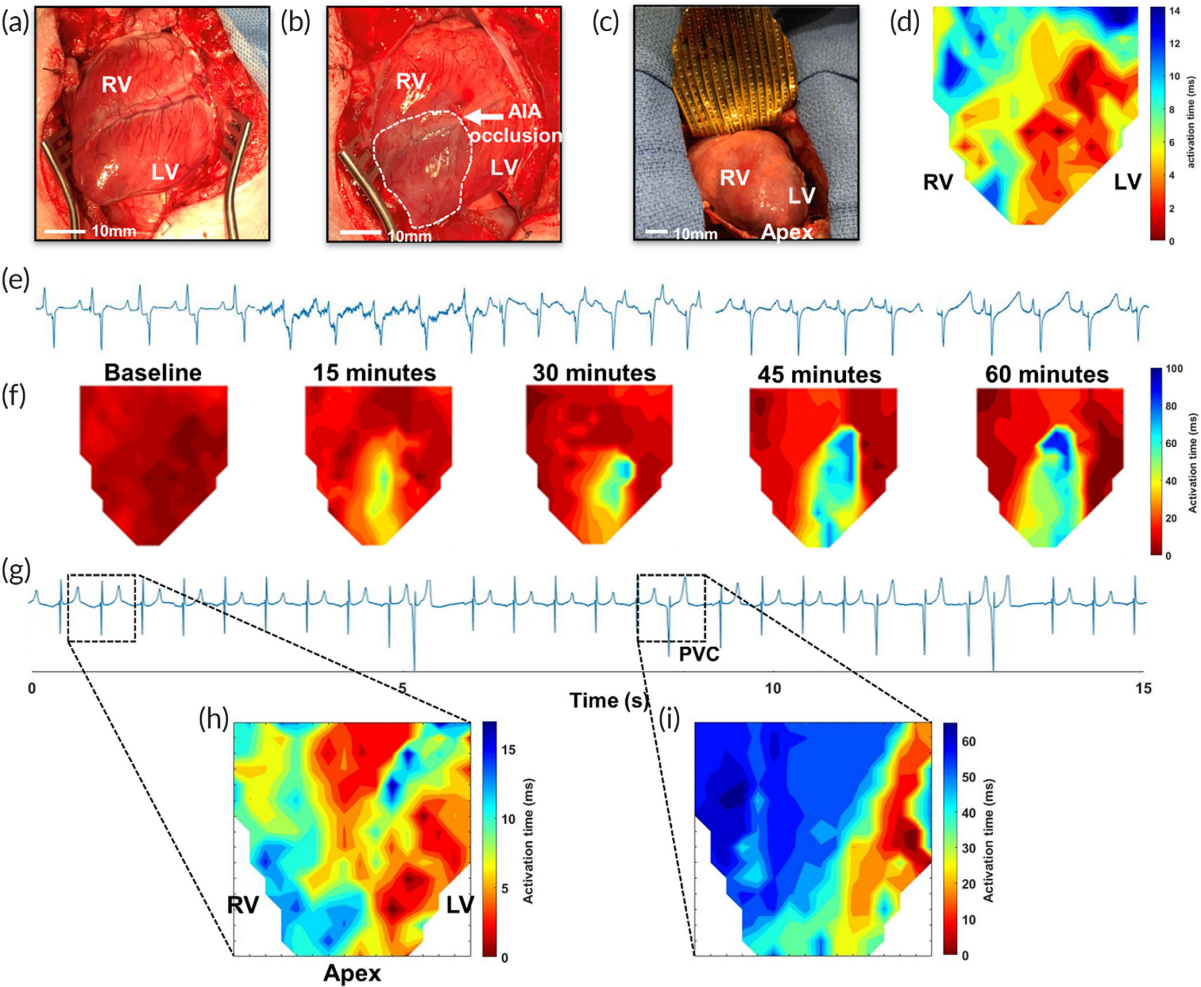
Serial mapping of the ventricular epicardium during myocardial ischemia and beat‐to‐beat capture of premature ventricular complexes. (a) Representative optical images of the anterior right ventricle (RV) and left ventricle (LV) after median sternotomy. (b) The anterior interventricular artery (AIA) was permanently ligated with suture and ischemia confirmed by visible blanching of the ischemic area at risk (white dotted area). (c) Serial ventricular mapping of the anterior RV and LV was performed using flexible ventricular arrays. (d) Baseline isochrone map of ventricular epicardial activation during sinus rhythm. (e) Changes to body surface ECG morphology were noted after AIA ligation with increasing evolution of ST‐elevation over 60 min. (f) Isochrone maps obtained at baseline, and every 15 min after AIA ligation, provide spatial information on the evolution of myocardial damage after ischemic injury. Mapping of the ventricular epicardium was also performed with identification of premature ventricular contractions (PVC). (g) PVCs were observed on surface ECG, while epicardial mapping captured beat‐to‐beat differences in wave propagation during (h), sinus rhythm and (i) PVCs, with the origin of the PVC originating from the lateral border of the LV.

Mapping of the ventricular chambers presents an additional challenge given the increased motion of the ventricular epicardial surface. Characterization of in vivo ventricular epicardial mapping was performed on Yorkshire swine who underwent median sternotomy to expose the anterior surface of the right and left ventricle (Figure 5a). In this experiment, the anterior interventricular artery (AIA) was ligated below the first diagonal branch to induce myocardial ischemia, Figure 5b. The flexible arrays enabled ventricular epicardial mapping of the anterior RV and LV surface at baseline and at 15 minutes intervals post‐AIA ligation (Figure 5c). Figure 5d shows baseline isochrone characterization prior to AIA ligation, with epicardial breakthrough emerging along the anterior left ventricular surface as previously described.^34^ The progression of ischemic injury following coronary artery ligation resulted in changes in body‐surface ECG morphology, as shown in Figure 5e. Whole‐chamber mapping demonstrated progressive slowing of conduction within the ischemic zone; isochrone maps at set time intervals illustrate the evolution of epicardial activation time within the ischemic region over a 60‐min time period, Figure 5f. This data validates the ability of the patient‐specific flexible devices to detect changes in action potential activation time over extended study periods with spatiotemporal reproducibility.

The same flexible arrays were used to localize the origin of premature ventricular complexes (PVC). Continuous epicardial mapping performed while a guidewire was introduced through the right internal carotid artery enabled capture of iatrogenic PVCs. Guidewire contact and subsequent irritation of the left ventricular myocardium induced PVCs, which were captured on body‐surface ECG, Figure 5g. Concomitant whole‐chamber epicardial mapping allowed for beat‐to‐beat capture and localization of the induced PVCs. Comparison between activation isochrones during sinus rhythm (Figure 5h) and PVC (Figure 5i) highlights the different origins of epicardial activation, with PVC activation occurring along the left ventricular border. The whole‐chamber array format allows for measurement of beat‐to‐beat changes in epicardial activation and precise localization of PVC sources, without the need for repositioning.^21^

### 2.7 Ex vivo Langendorff‐perfused human heart studies

**FIGURE 6:**
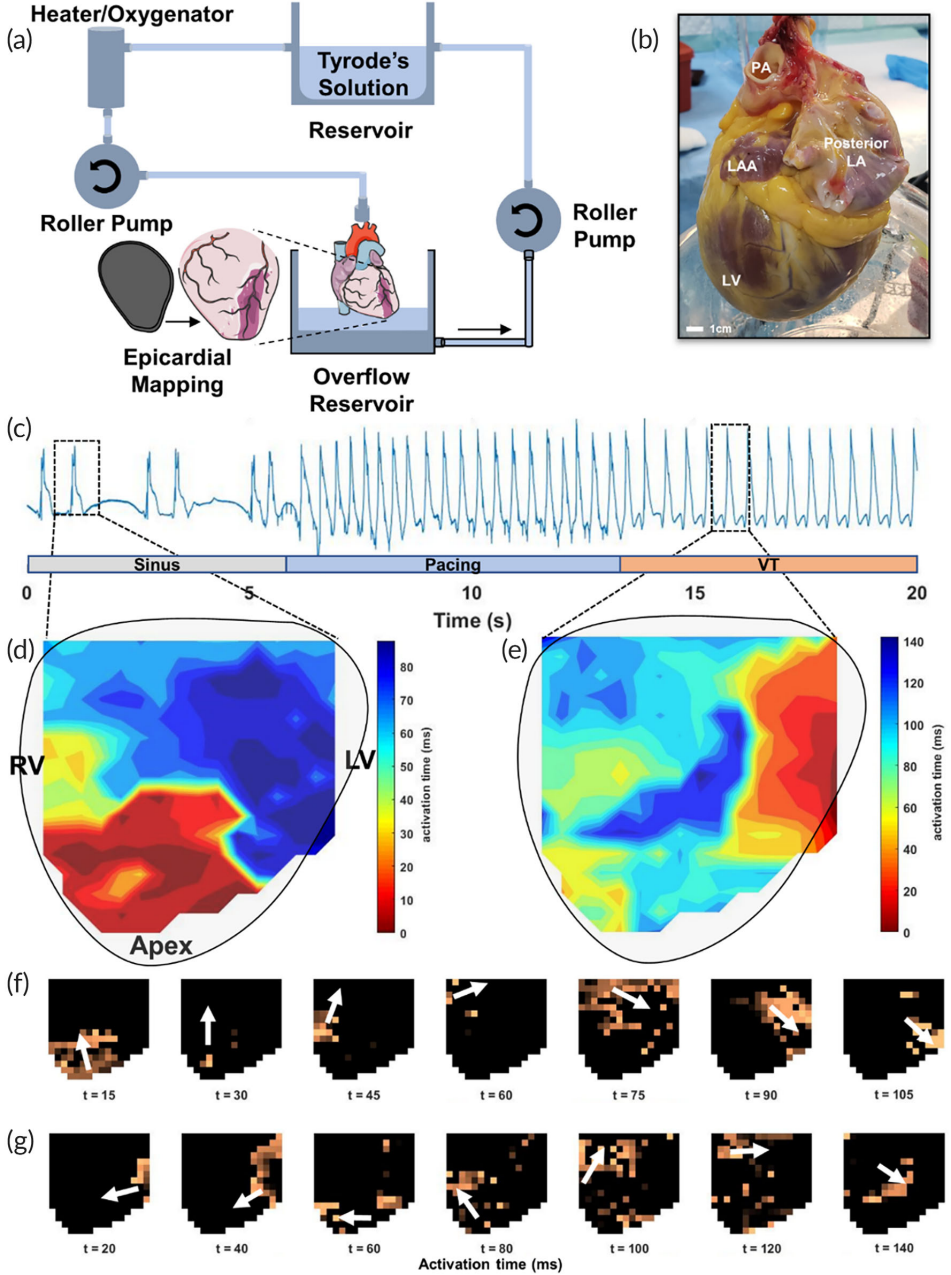
Ex vivo epicardial mapping of human‐dilated cardiomyopathy. (a) Donor hearts from patients with history of ventricular tachycardia (VT) were mounted on a Langendorff‐isolated heart perfusion system for epicardial mapping. (b) Representative optical image of a Langendorff‐perfused human heart depicts severe dilation of the LV. (c) Representative electrogram depicting capture of irregular baseline rhythm, extra stimulus pacing, and subsequent induced monomorphic VT. Ventricular mapping captured differences in epicardial wave propagation between (g) baseline and (h) VT rhythms. Spatial activation maps at select time intervals demonstrate differences in the direction of action potential propagation at (f) baseline originating from the ventricular apex, compared to (g) VT originating from the lateral border of the LV. LA, left atrium; LAA, left atrial appendage; LV, left ventricle; PA, pulmonary artery.

Validation of the diagnostic function of the 3D‐printed flexible arrays on human hearts was performed using a Langendorff‐perfused isolated heart system, Figure 6a. Human hearts were explanted from heart transplant recipients who consented to organ donation for research purposes, allowing for diagnostic mapping of pathologic hearts with known conduction disease. In particular, the flexible electrode arrays facilitated high spatial–temporal resolution capture of epicardial electrograms in a subset of patients with history of refractory ventricular tachycardia (VT). A major limitation and challenge of cardiac mapping during VT is hemodynamic intolerance of the induced arrhythmia, with <10% of the induced arrhythmias considered stable.^35^

An example of an explanted human heart with severe dilate cardiomyopathy, ejection fraction <20%, and history of refractory VT is shown in Figure 6b (and Video S2). Severe dilation of the LV was apparent, with wall motion abnormalities noted on reperfusion with the Langendorff system. Results of epicardial mapping performed over the anterolateral surface of the ventricle corresponds with an irregular junctional bigeminy rhythm at baseline. VT was induced through overdrive pacing delivered to the ventricular apex via the flexible arrays. External programmed stimulation successfully induced monomorphic VT; a representative electrogram highlights the induction of VT by programmed ventricular stimulation, Figure 6c. Beat‐to‐beat comparison between isochrones at baseline (Figure 6d) and VT (Figure 6e) demonstrates two distinct activation patterns before and after external pacing (Video S3). Spatial activation maps at selected time intervals illustrate differences in the origin of activation as they propagated along the ventricular epicardial surface. The activation map at baseline in Figure 6f, corresponds with activation along the ventricular apex. In contrast, the activation map indicates VT originating from the lateral border of the LV, Figure 6g. These results demonstrate the possibility of using whole‐chamber mapping arrays for the diagnosis and treatment of arrhythmias in humans.

We show here, as proof‐of‐concept, the ability for patient‐specific flexible arrays to quickly identify sites of VT origin. Importantly, whole‐chamber coverage of the ventricular surface eliminates the need for serial mapping of focal areas, which can be time‐consuming. Thus, the use of patient‐specific epicardial devices carries the promise of quick and efficient cardiac mapping of ventricular arrhythmias where time is often a priority in the face of unstable arrhythmias and risk of cardiogenic shock.

## 3 DISCUSSION

We describe workflows for the rapid production of flexible electrode arrays for whole‐chamber epicardial mapping. Complex epicardial geometries captured via conventional cardiac CT or MRI imaging can be optimized for 3D printing of patient‐specific mapping arrays. The approach can be applied broadly to other fields where accurate diagnosis can be difficult due to complex geometries.

We demonstrate the efficacy of using patient imaging to optimize electrode contact by capturing MRI specific geometric data. These workflows offer unique translatable opportunities where global coverage and optimization of electrode contact can improve the accuracy of diagnostics and therapeutics. The ability for the flexible arrays to deliver programmable extra stimulus pacing and RF energy enables concomitant execution of both diagnostic and interventional therapies, which offer the potential to customize treatment of atrial and ventricular arrhythmias.

The methods developed here can serve as a complement to existing techniques and provide more insight into cardiac electromechanical dynamics. Direct contact electrode arrays capable of performing whole‐chamber mapping offer a distinct advantage over current catheter‐based mapping systems, which are limited in coverage area and require sequential signal acquisition to construct global maps. Recent efforts to develop standalone epicardial mapping devices have demonstrated advancement to human trials; however, further work is needed to optimize approaches for mapping the complex and dynamic environment of the epicardial surface.^36^

In addition, flexible materials that combine various electronics and sensors have been successfully developed for the purpose of high‐resolution capture of epicardial and endocardial signals.^16^, ^17^, ^18^ Uniting these approaches with patient‐specific 3D‐printed substrates and a minimally invasive deployment may help promote translation of these materials into the surgical environment. When access to certain surfaces is difficult via traditional sternotomy or minimally invasive surgical approaches, these new thin and stretchable materials may benefit from attachment to patient‐specific 3D‐printed substrates for accurate deployment and positioning. Future iterations of these patient‐specific devices may be deliverable through various minimally invasive surgical pathways. Use of 3D‐printed shape memory polymers may enable minimally invasive deployment of flexible arrays via video‐assisted thoracoscopic surgical procedures and/or percutaneous subxiphoid access routes. In addition, flexible 3D‐printed endovascular arrays may be beneficial in cases where complex patient anatomy makes use of conventional catheters difficult. The engineering and development of endovascularly deployed 3D printed sensors would fit nicely into the current paradigm of electrophysiological mapping and ablation. Future studies into the thromboembolic risks and biocompatibility of such sensors will be crucial before embarking on endocardial mapping. Future developments can be further streamlined with direct 3D printing of electrode contacts into the flexible arrays.^37^

The field of cardiac surgery has just begun to realize the clinical and research potential of medical 3D printing. As arrhythmia surgery moves toward hybrid surgical approaches with cardiac surgeons and electrophysiologists, new tools will be needed to leverage their combined skillsets to treat complex cases. We have shown the utility of patient‐specific flexible arrays for applications relevant to diagnostic cardiac mapping and ablation therapeutics with RF energy. By leveraging existing clinical protocols, these patient‐specific flexible devices fit comfortably within the traditional clinical workflow prior to surgery. Short production timelines allow for personalized manufacture of custom devices immediately following acquisition of cardiac imaging. The capture of whole‐chamber, beat‐to‐beat data will in all likelihood prove to be beneficial, where the accurate localization of pathologic substrate is paramount to successful therapeutic intervention. Additional research is necessary to demonstrate that these techniques and tools may more effectively localize and treat arrhythmias.

## 4 MATERIALS AND METHODS

### 4.1 MRI acquisition and device fabrication

All experiments were conducted in accordance with the guidelines of the Institutional Animal Care and Use Committee (IACUC) of Stanford University. Yorkshire pigs weighing between 50 and 60 kg were imaged using an MRI (Signa HD× 3.0 T; GE Healthcare), with late gadolinium contrast. Images were loaded into a segmentation tool (ITK‐SNAP, www.itksnap.org) and the epicardial shell was isolated using a combined intensity‐based thresholding and manual segmentation approach.^22^ The segmentations were then assembled into a 3D reconstruction within ITK‐SNAP for export into CAD software (Fusion 360, AutoDesk, San Rafael, CA). Within the CAD software, the 3D reconstructions were smoothed, and a contoured shell was generated to mimic the epicardial surface and aligned according to landmark regions to ensure appropriate coverage of the whole chamber of interest. Key landmarks for registration included the superior vena cava, RAA and right ventricular border within the RA; the LAA in the left atrium; and the apex and right ventricular border for the left ventricle. The CAD model was then subdivided into equal‐spaced sections for appropriate electrode alignment. Next, epicardial shells were printed using a stereolithography 3D printer (Form3, Formlabs, Somerville, MA) for construction using flexible photopolymer resin (Flexible resin, FLFLGR02, Formlabs, Somerville, MA) with print resolutions of 25 and 50 μm, in the XY and Z directions respectively. After printing, the devices were cleansed in a solution of isopropyl alcohol for 20 min and then UV cured with 80.5 mW/cm^2^ of 365 nm fluorescent light for 60 min. Next, a 256‐channel custom‐designed flexible electrode array was mounted onto the epicardial shell. The flexible sensors were designed in‐house followed by prototype production (PCBcart, Hangzhou, China). The electrode array consisted of up to 256 individual gold‐coated electrodes measuring 1 mm in diameter, with 3–4 mm interelectrode spacing, dependent on total chamber size. The array was split into 16 individual sets of electrodes and aligned according to the subdivided sections identified within the CAD model, which allowed for flexibility and spacing to effectively cover the entire chamber of interest.

### 4.2 Device characterization

The impedance and performance of the flexible electronics were conducted using a benchtop impedance analyzer (Agilent 4294A, Santa Clara, CA, 40 Hz to 110 MHz). The electrodes were tested in both an unused condition and after a 1000‐flex cycle. The flexibility of the 3D‐printed device was tested using an electroactive stretch sensor (FlexSense, Parker, Cleveland, OH) embedded within the polymeric matrix of the device. Measurements were recorded based on the percent of stretch detected, with a baseline set at maximum elongation. Measurements were collected during active electrophysiological recording and exported for analysis. Force measurements were conducted by integrating 0–10 N capacitive force sensors (SingleTact, Los Angeles, CA) onto the posterior surface of the flexible arrays. Continuous force signal was output to a I2C/Arduino interface, and serial data were read into custom MATLAB software for analysis. Mechanical testing was characterized using a tension tester (Instron 5565, Norwood, MA), uniaxial tensile measurements were obtained from parts printed using a Form 3 stereolithography printer, 50 μm, and postcured with 80.5 mW/cm^2^ of 365 nm light for 60 min, tensile testing was performed using the ASTM D412 standard.

### 4.3 In vivo atrial mapping, pacing, and ablation in swine

Adult Yorkshire swine (50–60 kg) were used as healthy controls (*n* = 5), and for chronic AF modeling (*n* = 5). Chronic AF swine were implanted with two‐lead implantable cardiac pacemakers (Assurity MRI PM2272, Abbott, Chicago, IL), following a previously detailed protocol.^28^ The pacemaker leads were inserted through the right internal jugular vein and the leads implanted into the right interatrial septum and right atrial free wall. The animals were allowed to recover and then subjected to continuous threshold pacing at 350 bpm with amplitude of 10 mA for 6 weeks to induce AF. Once the pigs were confirmed to be in persistent AF after 6 weeks, the pacemaker was turned off, and the pigs were maintained in chronic AF for an additional 4 weeks before terminal electroanatomic mapping. Daily oral aspirin (81 mg) was administered for stroke prevention, and daily digoxin (0.25 mg with 0.25 mg increment every 2 weeks) was administered to prevent development of heart failure during high‐rate atrial pacing until the terminal procedure.

Prior to surgery, each animal was premedicated with Telazol, Ketamine, and Xylazine; intubated; and anesthetized with isoflurane. ECG and arterial pressure recordings were continuously monitored. A median sternotomy was performed and a pericardial sling created to expose the anterior surface of the myocardium. The epicardial mapping devices were placed directly onto the heart to collect electroanatomic mapping data. All electrogram data was recorded using a 256‐channel biopotential measurement system (2048 Hz sample rate, BioSemi, Amsterdam, Netherlands) and exported to MATLAB for data analysis. RF lesions were produced with a standard RF lesion generator (RFG‐6, Radionics, Burlington, MA) at a frequency of 500 kHz.

### 4.4 In vivo ventricular mapping in swine

Yorkshire swine (*n* = 5) underwent general anesthesia and median sternotomy as described above. The AIA was ligated below the level of the first diagonal artery to induce ischemia. Occlusion was confirmed by EKG changes and visual blanching of the anterior left ventricular wall. The mapping array was placed directly onto the anterior surface of the right and left ventricle to collect electroanatomic mapping data. Following completion of data acquisition, the animals were euthanized with an intracardiac injection of potassium chloride.

### 4.5 Ex vivo Langendorff heart perfusion

A Langendorff perfusion apparatus to support isolated whole‐heart preparations was constructed to conduct invasive electrophysiology studies. Deceased donor hearts (*n* = 5) were isolated from discarded organ transplantation procedures per protocol approved by the institutional review board. After removal, a heart was immersed in a cooled ice chest and transferred to the laboratory where it was then flushed with 2 L of chilled Tyrode solution and connected to the Langendorff perfusion.^38^, ^39^, ^40^ The perfusion apparatus consisted of a fluid reservoir, two in‐line roller pumps, a heater‐oxygenator, and a temperature‐controlled overflow reservoir (Figure 6a). The apparatus was equipped with in‐line temperature and intramyocardial temperature probes, in addition to pressure monitoring devices to ensure appropriate perfusate delivery and temperature equilibration. Once the heart achieved an intramyocardial temperature of 37°C, spontaneous beating and return of sinus rhythm was observed. An external Bloom programmable stimulator (Fischer Medical, Broomfield, CO) was used to pace the heart at a rate of approximately twice the diastolic threshold (2 mA with 2 ms pulse width). The external pacing site was modulated to a point on the RV, which subsequently induced VT. The ventricular epicardial mapping device was positioned across the left ventricle adjacent to the right ventricular border ending at the apex to ensure adequate coverage. Mapping was performed for three continuous cycles of 60 s to evaluate ventricular function.

### 4.6 Data analysis

Data from all channels were processed in a custom‐designed MATLAB program (Mathworks, Natick, MA). Unipolar electrograms were bandpass filtered (1–400 Hz). Atrial electrograms were preprocessed, and far field ventricular QRS‐T signaling was subtracted before atrial analysis. Activation times were determined by (d*V*/d*t*)_max_, and manually reviewed and edited for accuracy. Channels with poor contact or noise were removed and data interpolated. Isochronal contour maps were created by dividing total activation time into 15 equal time segments. Resultant isochrone maps depicted activation times of the entire mapped area, with red indicating early activation and blue indicating late activation. After bandpass filtering, power spectrum analysis was performed with additional rectification and Hanning windowing. The frequency band with the highest strength was called the DF, and DF with regularity indices below 0.2 were discarded. Final isochrone activation maps were interpolated onto 2D reconstructions of epicardial mapping probes to aid in visual reconstruction of electroanatomic maps.

## AUTHOR CONTRIBUTIONS

**Terrence Pong:** Conceptualization (lead); data curation (lead); formal analysis (lead); methodology (equal); writing – original draft (lead). **Kevin J Cyr:** Data curation (equal); formal analysis (equal); investigation (equal). **Cody Carlton:** Data curation (supporting). **Joy Aparicio‐Valenzuela:** Data curation (supporting); investigation (supporting). **Hanjay Wang:** Data curation (equal). **Meghedi Babakhanian:** Data curation (equal); investigation (equal); methodology (equal). **Alessandro Maiuolo:** Data curation (supporting). **Haley Lucian:** Data curation (supporting). **Paul J Wang:** Funding acquisition (supporting). **Joseph Y Woo:** Funding acquisition (supporting). **Anson M Lee:** Conceptualization (lead); funding acquisition (lead).

## FUNDING INFORMATION

This work was partly supported by a Stanford Bio‐X Interdisciplinary Initiatives Seed Grant and Cardiovascular Institute Seed Grant. Part of this work was performed at the Stanford Nano Shared Facilities (SNSF), supported by the National Science Foundation under award ECCS‐2026822. Animal experiments were performed in Stanford shared facilities supported by National Institutes of Health S10RR029020–01.

## CONFLICT OF INTEREST STATEMENT

The authors declare no conflicts of interest.

### PEER REVIEW

The peer review history for this article is available at https://www.webofscience.com/api/gateway/wos/peer‐review/10.1002/btm2.10575.

## Supporting information


**Supplemental Video S1.** Left atrial mapping via medial sternotomy.

**Supplemental Video S2.** Langendorff perfused human heart.

**Supplemental Video S3.** Langendorff human heart spatial activation map.

## Data Availability

The data that support the findings of this study are available on request from the corresponding author. The data are not publicly available due to privacy or ethical restrictions.
